# The Knockdown of Nrf2 Suppressed Tumor Growth and Increased the Sensitivity to Lenvatinib in Anaplastic Thyroid Cancer

**DOI:** 10.1155/2021/3900330

**Published:** 2021-09-04

**Authors:** Zhongqin Gong, Lingbin Xue, Minghui Wei, Zhimin Liu, Alexander C. Vlantis, C. Andrew van Hasselt, Jason Y. K. Chan, Dongcai Li, Xianhai Zeng, Michael C. F. Tong, George G. Chen

**Affiliations:** ^1^Department of Otorhinolaryngology, Head and Neck Surgery, The Chinese University of Hong Kong, Prince of Wales Hospital, Hong Kong; ^2^Department of Head & Neck Surgery, Cancer Hospital Chinese Academy of Medical Sciences, Shenzhen Center, China; ^3^Department of Biochemistry and Molecular Biology, Faculty of Basic Medical Sciences, Chongqing Medical University, Chongqing, China; ^4^Shenzhen Key Laboratory of ENT, Institute of ENT & Longgang ENT Hospital, Shenzhen, China; ^5^Shenzhen Research Institute, The Chinese University of Hong Kong, Shenzhen, Guangdong, China

## Abstract

Papillary thyroid cancer can dedifferentiate into a much more aggressive form of thyroid cancer, namely into anaplastic thyroid cancer. Nrf2 is commonly activated in papillary thyroid cancer, whereas its role in anaplastic thyroid cancer has not been fully explored. In this study, we used two cell lines and an animal model to examine the function of Nrf2 in anaplastic thyroid cancer. The role of Nrf2 in anaplastic thyroid cancer was investigated by a series of functional studies in two anaplastic thyroid cancer cell lines, FRO and KAT-18, and further confirmed with an *in vivo* study. The impact of Nrf2 on the sensitivity of anaplastic thyroid cancer cells to lenvatinib was also investigated to evaluate its potential clinical implication. We found that the expression of Nrf2 was significantly higher in anaplastic thyroid cancer cell line cells than in papillary thyroid cancer cells or normal control cells. Knockdown of Nrf2 in anaplastic thyroid cancer cells inhibited their viability and clonogenicity, reduced their migration and invasion ability *in vitro*, and suppressed their tumorigenicity *in vivo*. Mechanistically, knockdown of Nrf2 decreased the expression of Notch1. Lastly, knockdown of Nrf2 increased the sensitivity of anaplastic thyroid cancer cells to lenvatinib. As knockdown of Nrf2 reduced the metastatic and invasive ability of anaplastic thyroid cancer cells by inhibiting the Notch 1 signaling pathway and increased the cancer cell sensitivity to lenvatinib, Nrf2 could be a promising therapeutic target for patients with anaplastic thyroid cancer.

## 1. Introduction

Anaplastic thyroid cancer (ATC) is an aggressive form of thyroid cancer representing approximately 1% of thyroid cancers [1, 2]. ATC patients usually do not meet surgical treatment criteria at diagnosis and do not respond to chemotherapy or targeted therapies. Therefore, it accounts for the majority of thyroid cancer deaths [3]. The median overall survival for ATC is only 4 months from time of diagnosis, and its disease-specific mortality is almost 100% [4]. This dire situation indicates that the development of new treatment strategies for ATC is urgently needed.

Oxidants are increased in patients with thyroid cancer [5], suggesting that oxidative stress is a risk factor or a pathological element in thyroid cancer. Nuclear factor erythroid-derived 2-like 2 (NFE2L2), also known as Nrf2, is a transcription factor that can regulate multiple antioxidant and detoxifying enzymes [6]. In fact, Nrf2 has been implicated to be a mediator of thyroid cancer cell resistance to proteasome inhibitors, suggesting that Nrf2 may play an oncogenic role in thyroid cancer [7, 8]. In addition, Ziros et al. showed that Nrf2 was commonly activated in papillary thyroid cancer (PTC) [9]. A high expression of Nrf2 indicates a poor prognostic feature in PTC [10], supporting evidence that the activation of Nrf2 is an oncogenic event in PTC. The oncogenic function of Nrf2 has been further confirmed by inhibitory experiments in which the inhibition of Nrf2 promotes the antitumor effect of *Pinelliae rhizome* in PTC [11].

These studies all suggest that Nrf2 may be a potential therapeutic target in the treatment of thyroid cancer. However, the role of Nrf2 in ATC remains unclear. In this study, we evaluate the function of Nrf2 by using ATC cells and an animal model, aiming to provide evidence for the development of Nrf2 as a therapeutic target for ATC.

## 2. Materials and Methods

### 2.1. Cell Culture

Two differentiated thyroid cancer (DTC) cell lines, BCPAP and WRO, two ATC cell lines, FRO and KAT-18, and a normal control cell line Nthy-ori-3.1 were maintained in DMEM (Gibco, Life Technologies) medium supplemented with 10% (*v*/*v*) heat-inactivated fetal bovine serum (FBS, Gibco, Life Technologies). All cell lines were cultured as monolayers at 37°C in a 5% CO_2_ humidified atmosphere.

### 2.2. Production of Lentivirus for the Knockdown of Nrf2

Two Nrf2 shRNA plasmids (tet_pLKO.1_puro_shNRF2#1 and tet_pLKO.1_puro_shNRF2#2) were donated by Kevin Janes (Addgene plasmid #136584 and 136585). The corresponding control plasmid tet-pLKO.1_puro was donated by Dmitri Wiederschain (Addgene plasmid # 21915). Lentiviral preparations were generated by transient transfection of HEK-293T (Invitrogen) cells by using Lipofectamine 3000 (Invitrogen) with the Nrf2-shRNA/Nrf2-shctrl plasmid (2 *μ*g), the psPAX2 plasmid (1.5 *μ*g), and the pMD2.G plasmid (0.5 *μ*g). The cells were transfected with the lentivirus and selected by puromycin at 2 *μ*g/ml for two weeks. The knockdown efficiency was determined by Western blot.

### 2.3. Western Blot

The anti-Nrf2 (ABclonal, 1 : 2000), anti-Cyclin B1 (Cell Signaling, 1 : 2000), anti-E-cadherin (Cell Signaling, 1 : 2000), anti-Slug (Santa Cruz, 1 : 1000), anti-OCT4 (Cell Signaling, 1 : 2000), anti-Notch1 (Cell Signaling, 1 : 1000), anti-HES-1 (Cell Signaling,1 : 1000), and anti-GAPDH (Santa Cruz, 1 : 5000) were used as primary antibodies. A 1 : 20000 dilution of HRP-linked anti-IgG (Cell Signaling) was used as the secondary antibody. The enhanced chemiluminescent substrate and Western blot film plates from Kodak (Rochester, NY, USA) or Chemidoc MP (Bio-Rad) were used to detect protein levels on the membranes. The density of bands was quantified by ImageJ (National Institutes of Health).

### 2.4. Cell Proliferation Assay

An MTT kit (Sigma) was used to assay cell proliferation. Nrf2-knocked down FRO and KAT-18 cells and their corresponding control cells were seeded into 96-well plates at a density of 3000/well and cultured for different periods (24 h, 48 h, and 72 h). 10 *μ*l 5 mg/ml MTT was added to each well, and the optical density at 570 nm (OD570) was determined after 4 hours of incubation. The cell viability was represented by the OD570.

### 2.5. Migration and Invasion Assays

Approximately 5 × 10^3^ cells were suspended in 200 *μ*l of serum-free media and cultured in the upper chamber (8 *μ*m pore size, Millipore) for the migration assay. For the invasion assay, the chamber was coated with 20% Matrigel (Sigma) 24 hours before culturing cells. Approximately 1 × 10^4^ cells were suspended in 200 *μ*l of serum-free media and cultured in the upper chamber (8 *μ*m pore size, Millipore), and 800 ml of the cell growth medium was added to the lower chambers. After a 48-hour incubation, the cells that had migrated or invaded through the membrane from the upper chamber to the lower chamber were fixed by 4% paraformaldehyde and stained with 0.1% crystal violet (Sigma). The cells below the chamber surface were photographed, and the cells counted in three random fields.

### 2.6. Colony Formation Assay

A thousand cells were seeded into a 6-well plate for 10 days and the colonies stained with 0.1% crystal violet (Sigma) and counted with by ImageJ (National Institutes of Health).

### 2.7. Spheroid Formation Assay

Five thousand cells were seeded in Ultra-Low Attachment 6-well plates (Corning) with Cancer Stem Premium cell culture media (ProMab biotechnologies). Cells were grown for one week.

### 2.8. Apoptosis Assay

Apoptosis was detected by flow cytometry using the apoptosis assay kit (ab-176749 Abcam) as we have previously described [12].

### 2.9. Dual-Luciferase Reporter Assay

FRO and KAT-18 cells were transfected or cotransfected with HES1 promoter, Nrf2, Notch1 intracellular domain (NICD1), and the vector plasmids, as indicated in the figures. Cells were collected 48 h after transfection, and luciferase activities were detected by the dual-luciferase reporter assay kit (Promega, Fitchburg, WI). The luciferase activity was normalized to the control Renilla.

### 2.10. Coimmunoprecipitation

After HEK293T was transfected or cotransfected with plasmids, the nuclear protein was extracted for immunoprecipitation [13]. The ANTI-FLAG® M2 Affinity Gel was added into the lysis and incubated with continuing slow rotation at 4°C overnight. After washing and denatured, the samples were used for western blot analysis.

### 2.11. *In Vivo* Xenograft Assay

Female nude mice (4–6 weeks old, weighing 16–20 g) were provided by the Laboratory Animal Service Center of The Chinese University of Hong Kong. The mice had free access food and water on a 12 h light-12 h dark cycle. The nude mice were injected subcutaneously with 1 × 10^6^ KAT-18-shNrf2#1 (*n* = 5), KAT-18-shNrf2#2 (*n* = 4), and KAT-18 control cells (*n* = 5), respectively. The tumor size was measured every three days by a micrometer. The tumor volume was calculated by length × width × width/2. The mice were humanely euthanized 11 days after the tumor cells were transplanted with an overdose of anesthetic, and their tumors were collected for further investigation. All experimental procedures were approved by the Animal Ethics Committee of the Chinese University of Hong Kong.

### 2.12. IHC Staining

The tumor formed in the mouse model was used for the IHC staining study. The staining assay was performed according to the standard protocol on five-micrometer formalin-fixed paraffin sections. After staining, a pathologist and an investigator blind to the study design scored the staining intensities according to an immunoreactive score (IRS) system [14]. The primary antibodies for IHC are anti-Nrf2 (ABclonal, 1 : 200), anti-Ki-67 (Santa Cruz, 1 : 200), anti-Notch1 (Cell Signaling, 1 : 100), anti-c-Myc (Santa Cruz, 1: 100), and anti-Slug (Santa Cruz, 1 : 100).

### 2.13. IC50 Determination

Cells were treated with lenvatinib at different concentrations for 24 hours, and the cell viability was determined by MTT assay. The half maximal inhibitory concentration (IC50) values were calculated by an IC50 calculator (AAT Bioquest, http://aatbio.com/tools/ic50-calculator).

### 2.14. Statistical Analysis

In this study, the continuous data were presented as the median ± SD, and the discrete variables were presented as absolute values with their relative frequencies. Post hoc tests were performed. The Spearman correlation was used to evaluate the correlation between the expression of Nrf2 and other molecules. All tests were done with SPSS 22.0. *P* < 0.05 was considered statistically significant.

## 3. Results

### 3.1. The Knockdown of Nrf2 Inhibited ATC Cells Proliferation, Migration, and Invasion

**Figure 1:**
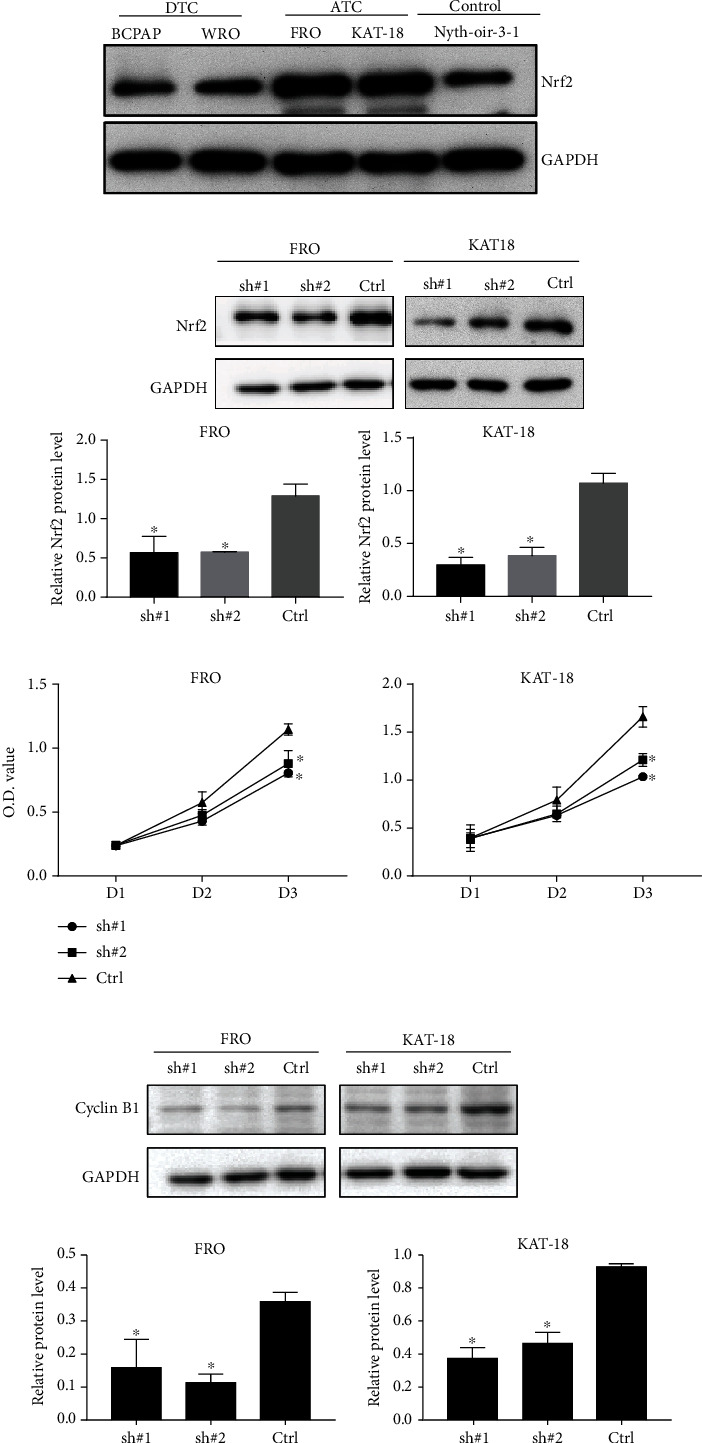
The knockdown of Nrf2 inhibited proliferation of FRO and KAT-18 cells. (a) The expression of Nrf2 in DTC, ATC, and control cells. (b) The stable knockdown of Nrf2 in FRO and KAT-18 cells after lentivirus transfection. (c) The knockdown of Nrf2 inhibited the proliferation of FRO and KAT-18 cells on MTT assay. Data are shown as mean ± SD, ^∗^*P* < 0.05. (d) The knockdown of Nrf2 suppressed the expression of cell cycle marker cyclin B1 in both FRO and KAT-18 cells, ^∗^*P* < 0.05.

To examine the effect of Nrf2 on the proliferation of ATC cells, we first selected two ATC cell lines with a high Nrf2 expression, FRO and KAT-18, which have unique genetic profiles [15] (Figure 1(a)). We silenced Nrf2 in the FRO and KAT-18 cells using a lentivirus shRNA plasmid. 2 *μ*g/ml puromycin was used to select stable cells. The inhibition of Nrf2 by its shRNA was verified by Western blot (Figure 1(b)). Compared to control cells, the MTT assay showed that the knockdown of Nrf2 significantly decreased the proliferation of FRO and KAT-18 cells (Figure 1(c)). The Western blot results showed that the knockdown of Nrf2 suppressed the expression of cell growth markers cyclin B1 in FRO and KAT-18 cells (Figure 1(d)).

**Figure 2:**
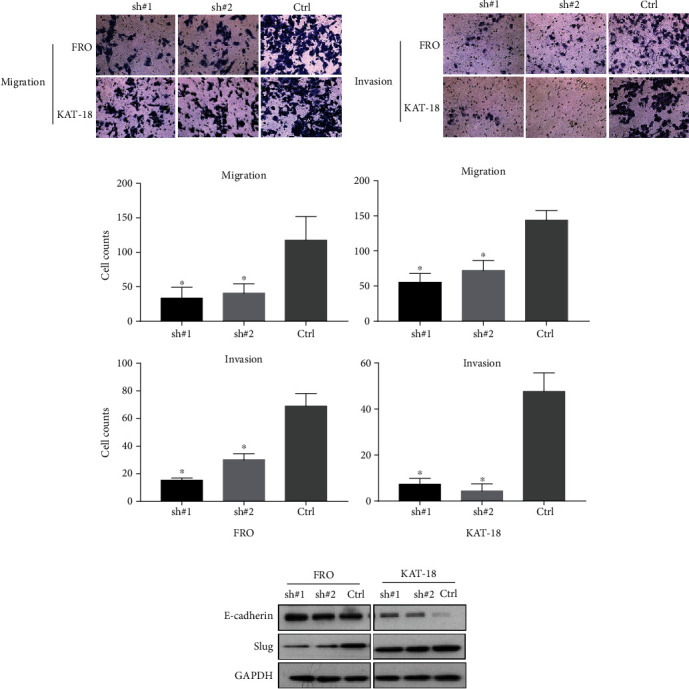
The knockdown of Nrf2 inhibited the aggressive properties of FRO and KAT-18 cells. (a) The knockdown of Nrf2 inhibited the migration ability of FRO and KAT-18 cells by transwell migration assay. (b) The knockdown of Nrf2 reduced the ability of invasion of FRO and KAT-18 cells by transwell invasion assay. (c) The histogram showed the numbers of migration and invasive cells, ^∗^*P* < 0.05. (d) The knockdown of Nrf2 inhibited the expression of Slug but increased the expression of E-cadherin in FRO and KAT-18 cells, suggesting that the knockdown of Nrf2 inhibited EMT in ATC cells.

To study the effect of Nrf2 on the migration and invasion ability of ATC cells, transwell migration and invasion assays were performed. Compared to control cells, the knockdown of Nrf2 inhibited the migration of FRO and KAT-18 cells (Figures 2(a) and 2(c)). Similar to the migration assay results, the invasion assay results showed that the knockdown of Nrf2 inhibited the invasive ability of FRO and KAT-18 cells (Figures 2(b) and 2(c)). Next, we examined the effect of Nrf2 on the expression of epithelial-mesenchymal transition (EMT) markers by Western blot. The results showed that after downregulation of Nrf2 in FRO and KAT-18 cells, the epithelial marker (E-cadherin) increased, and the EMT-related transcription factor Slug decreased (Figure 2(c)). These results suggest that the knockdown of Nrf2 exhibits an inhibitory role in ATC cells.

### 3.2. Downregulation of Nrf2 Inhibited Stemness and Induced Apoptosis in ATC Cells

**Figure 3:**
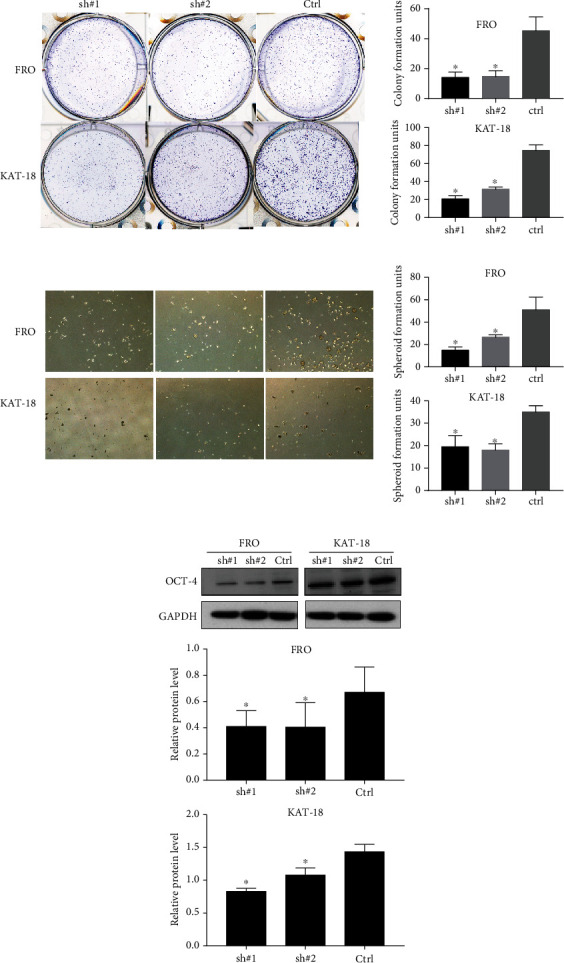
The knockdown of Nrf2 inhibited the stemness of ATC cells. (a) The number of colonies formed by shNrf2 cells was reduced compared to control cells in FRO and KAT-18 cells, ^∗^*P* < 0.05. (b) The knockdown of Nrf2 inhibited the spheroid formation of FRO and KAT-18 cells, ^∗^*P* < 0.05. (c) The knockdown of Nrf2 reduced the expression of stemness marker OCT-4 in FRO and KAT-18 cells, ^∗^*P* < 0.05.

To examine the effect of Nrf2 on the stemness of ATC cells, the colony formation assay and the spheroid formation assay were performed. The colony formation assay results showed that fewer colonies were formed in the Nrf2-knockdown FRO and KAT-18 cells (Figure 3(a)). We observed similar results in the spheroid formation assay. Compared to the control cells, the spheres were much smaller in the Nrf2-knockdown cells (Figure 3(b)). The Western blot results showed that knockdown of Nrf2 inhibited the expression of stemness markers OCT-4 (Figure 3(c)).

We next examined the effect of Nrf2 on the apoptosis of ATC cells by flow cytometry. The results showed that there were more apoptotic cells in FRO-shNrf2#1 (39.5% ± 0.31) and FRO-shNrf2#2 (36.1 ± 0.26) than control cells (19.4 ± 0.58) (Supplementary Fig. S1A). Similar results were observed in KAT-18 cells. The apoptotic populations in KAT-18 shNrf2#1 (40% ± 0.44) and KAT-18 shNrf2#2 (31.2 ± 0.72) were much higher than that in control cells (19.3 ± 0.04) (Supplementary Figure S1B).

### 3.3. Nrf2 Exerted Its Function in ATC Cells via the Notch1 Pathway

**Figure 4:**
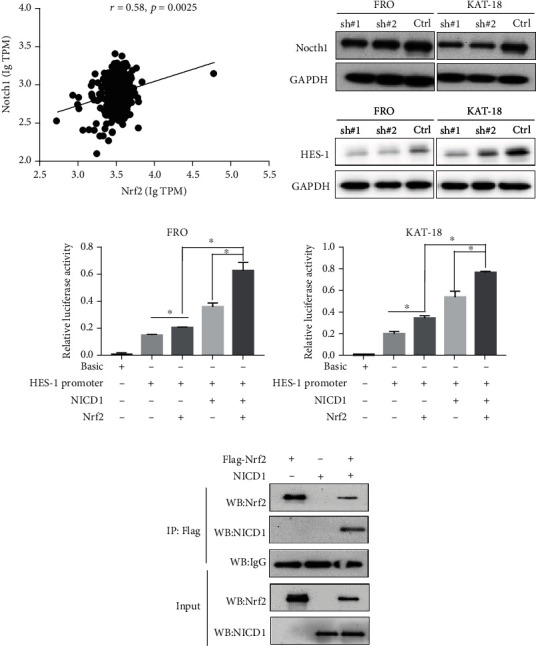
Nrf2 interacted with Notch1 to mediate the expression of HES-1 in ATC cells. (a) There is a positive correlation between Nrf2 and Notch1 in thyroid cancers (*r* = 0.58, *p* < 0.05). (b) The knockdown of Nrf2 reduced the expression of Notch1 and Notch1 target Hes-1 in FRO and KAT-18 cells. (c) Nrf2 was directly or synergistic with NICD1 to promote the HES-1 promoter activity in FRO and KAT-18 cells by luciferase assay, ^∗^*P* < 0.05. (d) Nrf2 interacted with NICD1 in HEK-293T cells by coimmunoprecipitation.

The Nrf2-Notch1 axis has been previously reported [16, 17]. However, its relationship in ATC is unclear. The Nocth1 signaling pathway has been shown to be involved in the pathogenesis of ATC [18]. We analyzed the correlation of Nrf2 and Notch1 with online database (http://gepia.cancer-pku.cn/detail.php) and (http://www.tsvdb.com/plot.html). The results showed that there is a positive correlation between Nrf2 and Notch1 in thyroid cancers (*r* = 0.58, *P* < 0.05) (Figure 4(a)). Our Western blot results showed that the knockdown of Nrf2 decreased the expression of Nocth1 and its target HES-1 in FRO and KAT-18 cells (Figure 4(b)). The luciferase assay showed that Nrf2 increased the promoter activity of HES-1. In addition, we observed a synergetic effect of Nrf2 and NICD1 in inducing the promoter activity of HES-1 when compared to Nrf2 or NICD1 (Figure 4(c)). Furthermore, the coimmunoprecipitation results showed that the Nrf2 interacted with the NCID1 (Figure 4(d)). These results suggest that Nrf2 might exert its functions via the Nocth1 pathway in ATC cells.

### 3.4. The Knockdown of Nrf2 Inhibited Tumor Growth *In Vivo*

**Figure 5:**
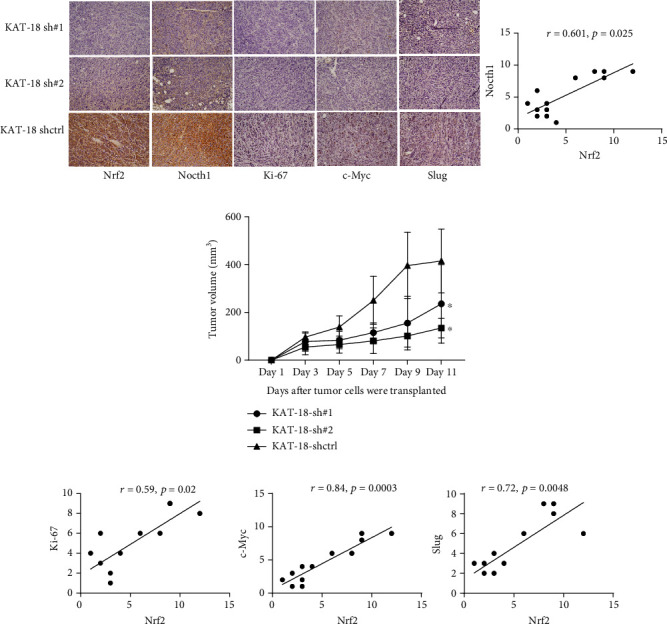
The knockdown of Nrf2 reduced the tumor volume in nude mice. (a) The representative image for tumor growth in nude mice and the detection of relevant proteins with IHC staining. (b) Subcutaneous tumor growth curve of KAT-18-shNrf2 in nude mice compared with the control. The tumor volume in the Nrf2 knockdown group was much smaller than the control (*P* < 0.05). (c) The Spearman correlation analysis of the correlation between Nrf2 and 4 other molecules, Notch1 (*r* = 0.601, *P* = 0.025), Ki-67 (*r* = 0.59, *P* = 0.02), c-Myc (*r* = 0.84, *P* < 0.001), and Slug (*r* = 0.72, *P* < 0.001).

To confirm the *in vitro* results, a xenograft assay was performed in nude mice. We found that the tumor volume formed by KAT-18-shNrf2#1 and KAT-18-shNrf2#2 was smaller than that formed by control cells (Supplementary Figure S2). The IHC representative images are shown in Figure 5(a). The tumor growth curve confirms that knockdown of Nrf2 inhibited tumor growth *in vivo* (Figure 5(b)). The IRS of Nrf2, Notch1, Ki-67, c-Myc, and Slug was decreased in shNrf2 groups than in control group (Supplementary Figure S3). In addition, we found that the expression of Nrf2 was positively correlated with the expression of Notch1 (*r* = 0.601, *P* = 0.025), Ki-67 (*r* = 0.59, *P* = 0.02), c-Myc (*r* = 0.84, *P* < 0.001), and Slug (*r* = 0.72, *P* < 0.001). These results are consistent with our *in vitro* results and suggest that Nrf2 plays an oncogenic role in ATC cells.

### 3.5. The Knockdown of Nrf2 Increased the Sensitivity to Lenvatinib

**Figure 6:**
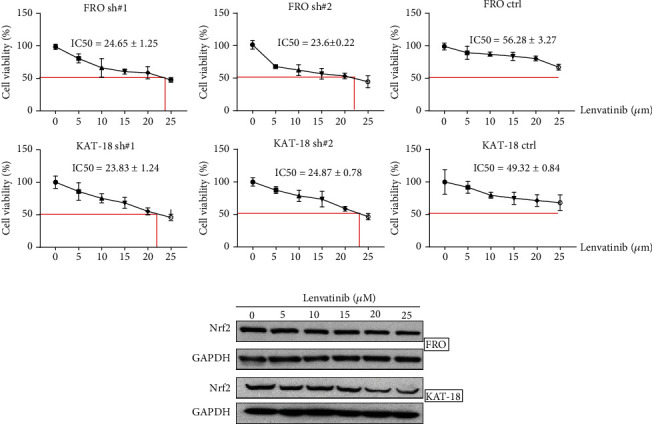
The knockdown of Nrf2 increased the sensitivity of lenvatinib in FRO and KAT-18 cells. (a) The 24 h IC50 of lenvatinib in FRO-shNrf2 and control cells was determined by MTT assay. The knockdown of Nrf2 decreased the IC50 value in FRO cells. A similar method was used to detect the IC50 of lenvatinib in KAT-18-shNrf2 and control cells, and the results showed that knockdown of Nrf2 decreased the IC50 value in KAT-18 cells. (b) Lenvatinib inhibits the expression of Nrf2 in FRO and KAT-18 in a dose-dependent manner.

To explore the clinical implications of Nrf2 on ATC cells, we evaluated the impact of Nrf2 on the effect of lenvatinib that has been used to treat patients with ATC [19–21]. We first determined the IC50. Cells with Nrf2 downregulation and control cells were treated with lenvatinib at different concentrations (0, 5, 10, 15, 20, and 25 *μ*M) for 24 hours. The results showed that the IC50 in FRO-shNrf2 and KAT-18-shNrf2 cells were less than 25 *μ*M, whereas the IC50 in control cells were 56.28 *μ*M and 49.32 *μ*M in FRO and KAT-18 cells, respectively (Figure 6(a)). We next examined the effect of lenvatinib on the expression of Nrf2 in FRO and KAT-18 cells. Cells were treated with lenvatinib at different concentrations for 24 hours, and the proteins were collected for Western blot. The results showed that lenvatinib inhibited the expression of Nrf2 in a dose-dependent manner in both FRO and KAT-18 cells. These results suggest that Nrf2 is a critical molecule in the lenvatinib-mediated suppression of ATC and that the knockdown of Nrf2 increased the sensitivity of ATC cells to lenvatinib.

## 4. Discussion

During the past decades, the incidence of thyroid cancer has increased substantially [2, 22, 23], resulting in it having a significant effect on public health. Most DTC cases respond to surgical combined radioiodine (RAI) therapy. However, there are 5-10% of thyroid cases with recurrence, developed to RAI refractory and advanced thyroid cancer [24].

Dabrafenib and trametinib have been used as targeted therapy in advanced or metastatic thyroid cancer with the BRAF V600E mutation, which has shown promise [25]. Recently, Arikan et al. [26] reported the rechallenge of a 52-year-old ATC patient with BRAF mutation with dabrafenib plus trametinib, and this is the first ATC case that respond to dabrafenib-trametinib reported in the literature. The BRAF mutation is the most common genetic alteration in PTC [27]. However, TP53 mutations are the most frequent in poorly differentiated thyroid cancer and ATC [28–30], suggesting that the application of dabrafenib and trametinib in ATC might be limited. Therefore, the discovery of novel therapeutic target for ATC is urgently needed.

Numerous studies have shown that ATC is dedifferentiated from DTC [31–33], enabling it to possess more aggressive properties, such as invasiveness and metastatic potential. Besides, the EMT process can lead to the dedifferentiation of DTC [34], and the stemness property may lead to drug resistance in thyroid cancer [35]. Therefore, the inhibition of such aggressive properties may be beneficial in the treatment of ATC. Nrf2 plays a central role in the growth advantage, metastatic property, and resistance to cancer therapies [36]. Our data showed that the expression of Nrf2 protein was higher in the ATC cells than that in DTC cells and normal control cells, suggesting that the inhibition of Nrf2 has a potential application in ATC therapy [37]. In addition, the knockdown of Nrf2 inhibited the migration and invasive ability, the expression of EMT markers, and the stemness of ATC cells, indicating that Nrf2 may be a promising target for the treatment of ATC.

Several studies have indicated that lenvatinib, a multikinase inhibitor which has been approved for the treatment of radioiodine refractory differentiated thyroid cancer, can generate particular benefits for ATC regardless of gene mutation status [21]. Although lenvatinib can effectively reduce the tumor burden, the survival benefits were doubtful [19]. A single-center, retrospective analysis claimed that in patients with ATC who had progressed on prior therapy, the addition of lenvatinib could improve survival duration and reduce tumor volume [20]. Our study showed that the knockdown of Nrf2 could enhance the sensitivity of ATC cells to lenvatinib, suggesting that the combination of shNrf2 and lenvatinib may generate a synergetic effect for the treatment of ATC.

It is known that the expression of Notch1 is higher in ATC than in PTC, and the knockdown of Notch1 can significantly reduce the proliferation and migration of ATC cells, but not PTC cells [18]. Our data has further connected the Notch1 pathway with Nrf2 by showing that the knockdown of Nrf2 decreases the expression of Notch1 and its target HES-1 in ATC cells. Moreover, the luciferase assay and coimmunoprecipitation assay results have demonstrated that Nrf2 and NICD1 can form a protein complex to increase the promoter activity of HES-1. Therefore, mechanically, our results have revealed a novel Nrf2-Notch1 axis in ATC.

## 5. Conclusion

In conclusion, the knockdown of Nrf2 inhibited tumor growth *in vitro* and *in vivo*. Cells with reduced expression of Nrf2 were more sensitive to the lenvatinib treatment. Our study provided evidence for developing Nrf2 as a novel therapeutic target for ATC.

## Data Availability

All data generated or analyzed during this study are included in this published article and its supplementary information files.

## Supplementary Materials

Supplementary Materials Figure S1: the inhibition of Nrf2 induced apoptosis in FRO and KAT-18 cells, ^∗^*P* < 0.05. Figure S2: the size of tumor formed by Nrf2 knockdown KAT18 cells was much smaller than the tumor formed by control cells. Figure S3: the immunoreactive scores (IRS) in tumor samples form xenograft mouse model. The expression of Nrf2, Nocth1, Ki-67, c-Myc, and Slug were significantly decreased in Nrf2 knockdown tumor samples than that in control samples, ^∗^*P* < 0.05.
