# Supplementary material for: The Knockdown of Nrf2 Suppressed Tumor Growth and Increased the Sensitivity to Lenvatinib in Anaplastic Thyroid Cancer
Source: Oxid Med Cell Longev. 2021 Sep 4;2021:3900330. doi: 10.1155/2021/3900330 (PMC8437598; doi:10.1155/2021/3900330)
Supplement: Supplementary Materials — Figure S1: the inhibition of Nrf2 induced apoptosis in FRO and KAT-18 cells, ∗P < 0.05. Figure S2: the size of tumor formed by Nrf2 knockdown KAT18 cells was much smaller than the tumor formed by control cells. Figure S3: the immunoreactive scores (IRS) in tumor samples form xenograft mouse model. The expression of Nrf2, Nocth1, Ki-67, c-Myc, and Slug were significantly decreased in Nrf2 knockdown tumor samples than that in control samples, ∗P < 0.05. [file 3900330.f1.docx]

**Supplementary figures**

**The knockdown of Nrf2 suppresses anaplastic thyroid cancer and increases its sensitivity to lenvatinib**

Zhongqin Gong^1*^, Lingbin Xue^1*^, Minghui Wei^2^, Zhimin Liu^3^, Alexander C Vlantis^1^, C Andrew van Hasselt^1^, Jason YK Chan^1^, Dongcai Li^4^, Xianhai Zeng^4^, Michael C. F. Tong^1,#^ , George G Chen^1,5#^


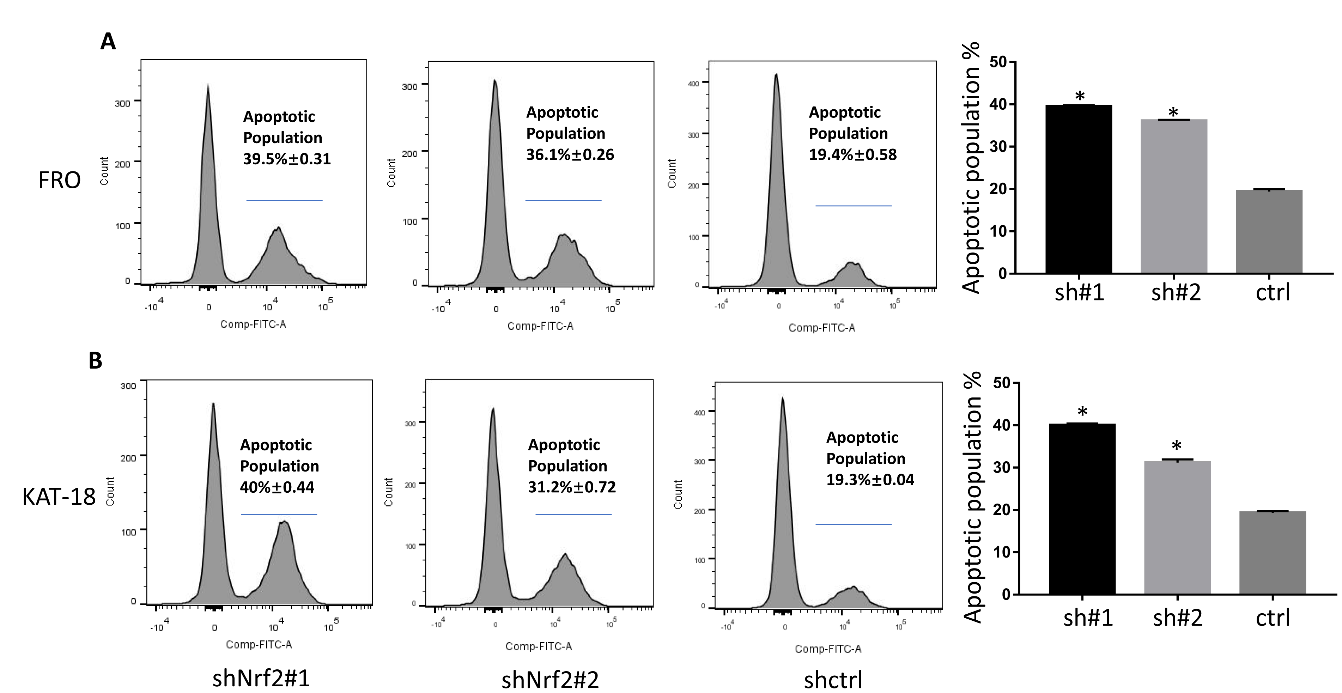


Fig S1. The inhibition of Nrf2 induced apoptosis in FRO and KAT-18 cells, *P<0.05.


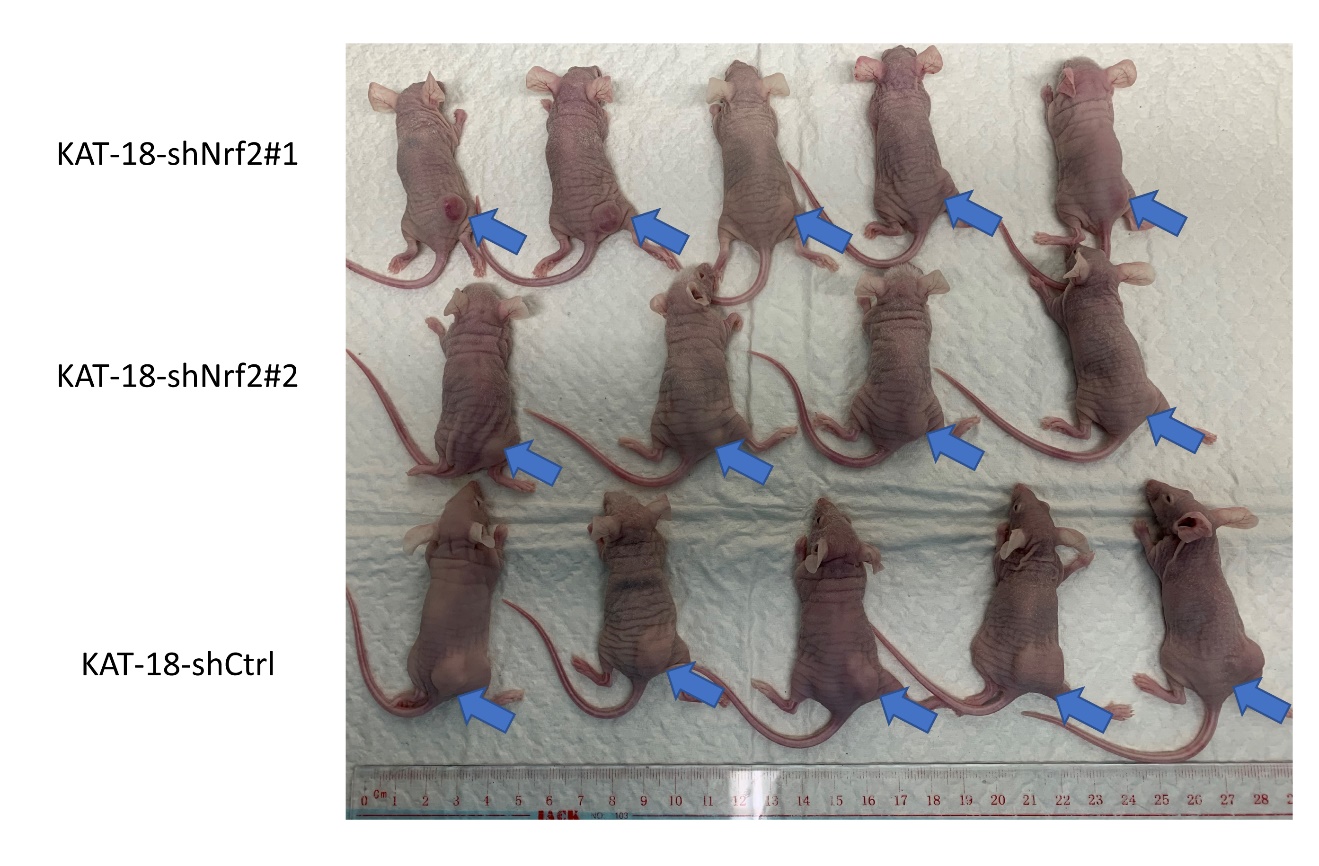


Fig S2. The size of tumor formed by Nrf2 knockdown KAT18 cells was much smaller than the tumor formed by control cells.


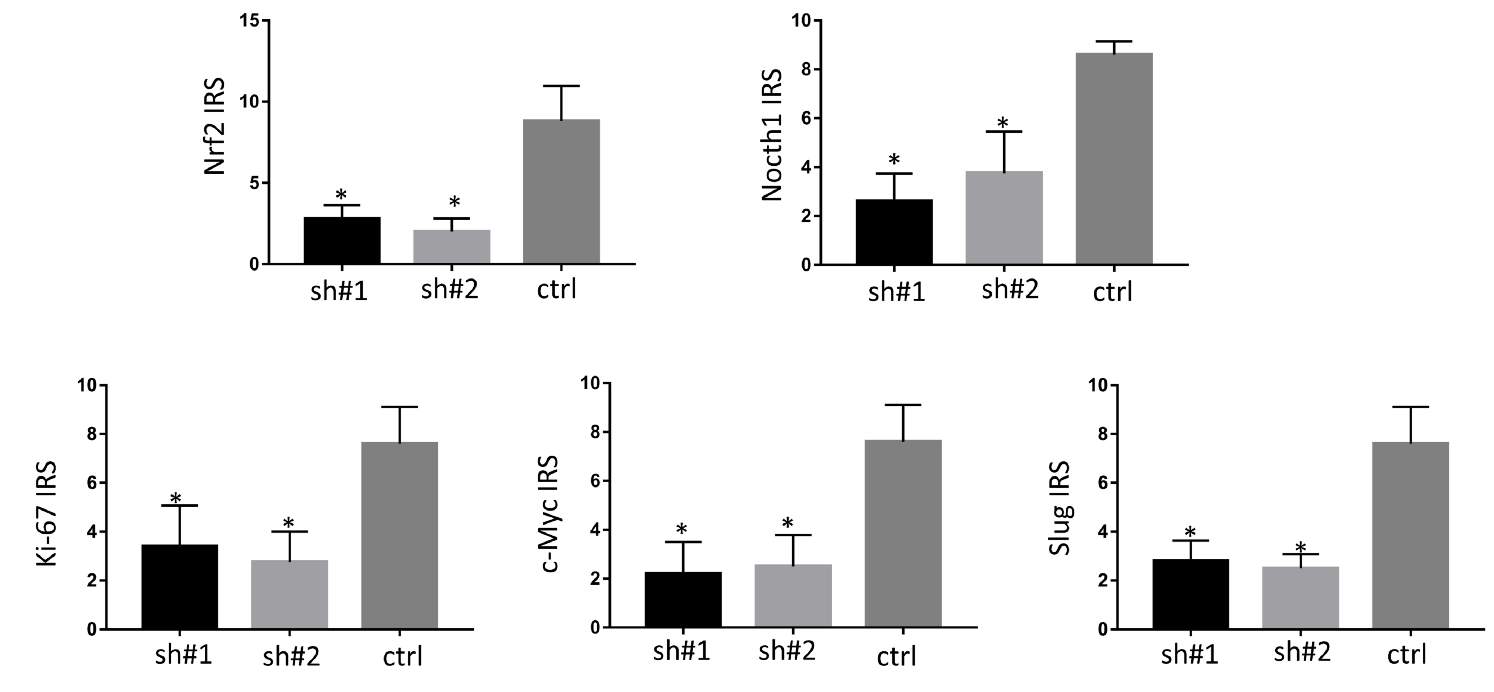


Fig S3. The immunoreactive scores (IRS) in tumor samples form xnograft mouse modle. The expression of Nrf2, Nocth1, Ki-67, c-Myc and slug were significantly decreased in Nrf2 knockdown tumor samples than that in control samples, *P<0.05.
